# Combined inflammatory-lipid index and tumor markers for predicting the spatial localization of lesions in early-stage non-small cell lung cancer

**DOI:** 10.3389/fonc.2025.1635315

**Published:** 2025-10-09

**Authors:** Zichen Yang, Shouxiang Zhao, Zhenting Cheng, Dengfeng Ge, Hao Ren, Jiankang Xu, Bin Zhang

**Affiliations:** ^1^ Nanjing University of Chinese Medicine, Nanjing, Jiangsu, China; ^2^ Department of Cardiothoracic Surgery, Affiliated Hospital of Nanjing University of Chinese Medicine, Jiangsu Province Hospital of Chinese Medicine, Nanjing, China

**Keywords:** NSCLC, spatial localization of lesions, predictive mode, tumor markers, inflammatory-lipid indices

## Abstract

**Objective:**

This study evaluated the predictive value of combined inflammatory-lipid indices and tumor markers in determining lesion localization in early-stage non-small cell lung cancer (NSCLC) and developed a predictive model.

**Methods:**

A retrospective analysis of 206 early-stage NSCLC patients was conducted from December 1, 2023, to September 30, 2024. Patients were grouped based on tumor location: upper lobe and lower lobe. Significant predictors were identified through univariate and multivariate logistic regression analyses, leading to the development of a nomogram. Predictive performance was assessed using the receiver operating characteristic (ROC) curve and area under the curve (AUC). Model calibration was evaluated with a calibration plot, and decision curve analysis (DCA) was utilized to assess the model’s relevance in clinical settings.

**Results:**

Among the 206 patients, 135 (65.53%) had upper lobe tumors, and 71 (34.47%) had lower lobe tumors. Significant differences were found in white blood cell (WBC) count, lymphocyte count, α-hydroxybutyrate dehydrogenase (α-HBDH), high density lipoprotein cholesterol (HDL) triglycerides, low-density lipoprotein cholesterol (LDL), total cholesterol, carcinoembryonic antigen (CEA), serum ferritin (SF), carbohydrate antigen 125 (CA125), carbohydrate antigen 153 (CA153), and carbohydrate antigen 199 (CA199) (all *p* < 0.05). Multivariate logistic regression identified WBC (OR: 1.46, 95% CI: 1.13–1.95, *p* = 0.007), a-HBDH (OR: 1.01, 95% CI: 1.00–1.03, *p* = 0.041), HDL (OR: 7.08, 95% CI: 1.50–36.16, *p* = 0.015), CEA (OR: 1.12, 95% CI: 1.02–1.23, *p* = 0.021), SF (OR: 1.01, 95% CI: 1.00–1.02, *p* = 0.020), CA153 (OR: 1.08, 95% CI: 1.00–1.16, *p* = 0.037), and CA199 (OR: 1.16, 95% CI: 1.07–1.27, *p* < 0.001) as independent risk factors for lower lobe tumor localization. An AUC of 0.806 was obtained for the nomogram (95% CI: 0.743–0.868), indicating good calibration, and showed favorable clinical utility based on decision curve analysis (DCA).

**Conclusion:**

WBC count, lymphocyte count, α-HBDH, HDL, CEA, SF, CA153, and CA199 are significant predictors of lesion localization in early-stage NSCLC. The developed nomogram, based on readily available clinical parameters, demonstrated strong predictive performance and may aid in individualized diagnosis and treatment planning. Further large-scale external validation is needed.

## 1 Introduction

Lung cancer ranks among the most frequently diagnosed types of cancer globally. It is classified into two primary subtypes: small cell lung cancer (SCLC) and non-small cell lung cancer (NSCLC), with NSCLC making up about 85% of cases. In China, an estimated 787,000 new NSCLC cases are diagnosed annually, representing 40% of the global burden, while the five-year survival rate remains as low as 19.7% (1). As public awareness increases and lung cancer screening programs are implemented, early-stage lung cancer detection has improved. The WHO categorizes NSCLC into three primary histopathological subtypes: adenocarcinoma, squamous cell carcinoma, and large cell carcinoma (2). Early detection of NSCLC is vital; however, due to the absence of dependable biomarkers, around 60% of patients are diagnosed at later stages (3). Studies suggest that the anatomical location of primary tumors in malignancies like esophageal cancer, colorectal cancer, and NSCLC correlates with prognosis (4, 5).Tumor location is a known prognostic factor in metastatic lung cancer. A significant link between tumor recurrence and lesions in the lower lobes of the lungs in NSCLC patients was found by Motono et al. (6). A multicenter retrospective analysis established tumor location as an independent predictor of survival in patients with completely resected pathologic stage I non-small cell lung cancer (NSCLC). This investigation identified that stage I NSCLC patients with lower lobe tumors experienced inferior survival outcomes compared to those with tumors located in the upper or middle lobes (7). A meta-analysis investigating tumor localization and survival in stage I-III non-small cell lung cancer demonstrated that upper lobe tumor location was associated with superior three-year survival, whereas lower lobe localization correlated with inferior survival outcomes (8). However, research on the spatial localization of primary lesions in NSCLC is limited. Identifying predictors for lesion location is therefore a crucial yet underexplored area.

## 2 Methods

### 2.1 Study cohort and design

This retrospective study, conducted at Jiangsu Provincial Hospital of Traditional Chinese Medicine, included early-stage NSCLC patients who underwent video-assisted thoracoscopic surgery (VATS) between December 1, 2023, and September 30, 2024.

Inclusion criteria as follows:

1. Participants were between 18 and 80 years of age;
2. Multiple pulmonary nodules on computed tomography (CT);
3. Undergoing VATS (wedge resection or lobectomy) with intraoperative frozen-section diagnosis confirming NSCLC, and had no history of radiotherapy, chemotherapy, or targeted therapy;
4. Postoperative pathological staging from IA to IIB was determined based on the 9th edition of the TNM system;
5. Each individual gave documented consent before taking part in the research.

Exclusion of participants was based on the following criteria:

1. History of other malignancies;
2. Severe dysfunction of the heart, liver, kidneys, or brain;
3. Metabolic or immune diseases, such as hyperthyroidism;
4. Active hematologic or infectious diseases;
5. Incomplete clinical data.

The Ethics Committee of Jiangsu Provincial Hospital of Traditional Chinese Medicine approved the study protocol. A flowchart of the study is presented in **Table 1**.

**Table 1:** Overall patients baseline (N = 206).

Variables	Number of patients(n=206)
Sex	
Male	66 (32.04%)
Female	140 (67.96%)
Age (year)	57.75 ± 12.55
Height (cm)	162.29 ± 7.12
Weight (kg)	63.11 ± 11.30
BMI (kg/m^2)	23.90 ± 3.62
Smoke	
No	180 (87.38%)
Yes	26 (12.62%)
Drink	
No	185 (89.81%)
Yes	21 (10.19%)
Hypertension	
No	135 (65.53%)
Yes	71 (34.47%)
Diabetes	
No	187 (90.78%)
Yes	19 (9.22%)
Coronary heart disease	
No	195 (94.66%)
Yes	11 (5.34%)
Focal location	
Upper lobe of lung	135 (65.53%)
Lower lobe of lung	71 (34.47%)

### 2.2 Definition of spatial localization of NSCLC lesions

Lesions in the upper lobes were defined as primary malignant tumors confined to the bronchial branches and peripheral lung tissues. The right upper lobe includes the apical (S1), posterior (S2), and anterior (S3) segments, while the left upper lobe comprises the apicoposterior (S1 + 2), anterior (S3), superior lingular (S4), and inferior lingular (S5) segments.

Lesions in the lower lobes were defined as primary malignant epithelial tumors confined to the lower lobe bronchi and distal lung tissues. The right lower lobe consists of the superior (S6), medial basal (S7), anterior basal (S8), lateral basal (S9), and posterior basal (S10) segments. The left lower lobe includes the superior (S6), anteromedial basal (S7 + 8), lateral basal (S9), and posterior basal (S10) segments (9).

### 2.3 Data collection

#### 2.3.1 Clinical data

The hospital’s medical record system served as the source for baseline clinical data, covering factors such as sex, age, height, weight, smoking habits (smoking at least one cigarette per day on average in the past year), alcohol intake (greater than 100 mL daily), comorbidities (hypertension, diabetes, and coronary artery disease), maximum tumor size, and lesion location.

#### 2.3.2 Laboratory data

Laboratory indicators included inflammatory markers (white blood cell count, neutrophils, lymphocytes, monocytes), liver and kidney function tests (aspartate aminotransferase, alanine aminotransferase, albumin, globulin), electrolytes (potassium, sodium, chloride, calcium), lipid profile (total cholesterol, triglycerides, high-density lipoprotein cholesterol (HDL), low-density lipoprotein cholesterol (LDL)), coagulation parameters (prothrombin time, fibrinogen, D-dimer), and tumor markers (carcinoembryonic antigen (CEA), serum ferritin (SF), carbohydrate antigen 125 (CA125), carbohydrate antigen 153 (CA153), carbohydrate antigen 199 (CA199)). These data were sourced from the Department of Clinical Laboratory at Jiangsu Provincial Hospital of Traditional Chinese Medicine.

### 2.4 Statistical analysis

Data analysis was conducted in R (v4.1.3). Categorical data are presented as counts (percentages), and continuous variables as means ± SD or medians with interquartile ranges. Comparisons between groups for continuous data utilized either the independent t-test or the Mann-Whitney U test, depending on distribution. Categorical data were analyzed using the chi-square test or Fisher’s exact test, as appropriate. Associations were quantified using odds ratios (ORs) along with 95% confidence intervals (CIs).

To assess factors related to lesion location, both univariate and multivariate logistic regression analyses were carried out. Variables with a p-value <0.05 in the univariate analysis were included in the multivariate logistic regression model. A nomogram was constructed based on significant predictors. The model’s efficacy was evaluated through the receiver operating characteristic (ROC) curve and corresponding area under the curve (AUC).To assess the model’s performance, calibration plots were generated to compare predicted versus actual outcomes, and DCA was used to determine its practical utility in clinical settings.

## 3 Results

### 3.1 Patients’ characteristics

This study included 206 patients: 66 males (32.04%) and 140 females (67.96%) (**Table 1**). Participants has a mean age of 57.75 ± 12.55 years, with a BMI of 23.90 ± 3.62 kg/m². A small proportion reported smoking (12.62%) and alcohol consumption (10.19%). Comorbidities included hypertension (10.19%), diabetes mellitus (34.47%), and coronary artery disease (5.34%). Tumors were located in the upper lobes in 135 patients (65.53%) and in the lower lobes in 71 (34.47%). Baseline parameters are shown in **Table 2**.

**Table 2:** Analysis of baseline characteristics.

Variables	Upper lobe of lung (N=135)	Lower lobe of lung (N=71)	P-value
Sex			0.238
Male	39 (28.89%)	27 (38.03%)	
Female	96 (71.11%)	44 (61.97%)	
Age (year)	58.05 ± 11.51	57.18 ± 14.39	0.661
Height (cm)	161.95 ± 7.40	162.94 ± 6.55	0.323
Weight (kg)	63.07 ± 11.66	63.19 ± 10.66	0.939
BMI (kg/m^2)	23.99 ± 3.80	23.72 ± 3.29	
Smoke			0.262
No	121 (89.63%)	59 (83.10%)	
Yes	14 (10.37%)	12 (16.90%)	
Drink			0.400
No	119 (88.15%)	66 (92.96%)	
Yes	16 (11.85%)	5 (7.04%)	
Hypertension			0.751
No	90 (66.67%)	45 (63.38%)	
Yes	45 (33.33%)	26 (36.62%)	
Diabetes			0.299
No	120 (88.89%)	67 (94.37%)	
Yes	15 (11.11%)	4 (5.63%)	
Coronary heart disease			0.193
No	130 (96.30%)	65 (91.55%)	
Yes	5 (3.70%)	6 (8.45%)	
Maximum tumor size (mm)	13.28 ± 7.11	13.63 ± 6.99	0.733

BMI, body mass index.

No notable differences were found between the upper and lower lobe groups in sex, age, BMI, smoking, alcohol use, hypertension, diabetes, coronary artery disease, or maximum tumor diameter (all *p* > 0.05). Laboratory parameters are shown in **Table 3**. Statistically significant differences were observed in WBC count (*p* = 0.001), lymphocyte count (*p* = 0.008), α-hydroxybutyrate dehydrogenase (α-HBDH) (*p* = 0.007), total cholesterol (*p* < 0.001), triglycerides (*p* = 0.027), HDL (*p* = 0.010), carcinoembryonic antigen (CEA; *p* = 0.031), serum ferritin (*p* = 0.004), CA125 (*p* = 0.005), CA153 (*p* = 0.017), and CA199 (*p* < 0.001).

**Table 3:** Analysis of laboratory test results.

Variables	Upper lobe of lung(N=135)	Lower lobe of lung(N=71)	P-value
WBC	5.03 ± 1.25	5.84 ± 1.84	0.001
NEUT	3.23 ± 1.15	3.48 ± 1.59	0.237
LYM	1.66 ± 0.52	1.89 ± 0.60	0.008
MONO	0.33 ± 0.10	0.35 ± 0.11	0.090
AST	20.64 ± 9.68	19.34 ± 7.63	0.292
ALT	22.39 ± 13.60	20.24 ± 14.20	0.296
ALB	41.29 ± 3.18	41.25 ± 2.42	0.928
GLB	24.32 ± 3.82	24.89 ± 3.41	0.275
ALP	74.34 ± 25.67	76.34 ± 23.10	0.571
GGT	27.99 ± 12.73	28.59 ± 15.83	0.781
CK	67.91 ± 27.04	75.63 ± 34.56	0.169
LDH	178.23 ± 30.34	184.51 ± 36.68	0.219
CKMB	13.06 ± 4.34	13.51 ± 4.90	0.585
α-HBDH	128.41 ± 19.81	137.83 ± 24.99	0.007
CRE	64.96 ± 15.04	65.49 ± 13.96	0.804
K	3.79 ± 0.30	3.83 ± 0.33	0.443
Na	141.30 ± 1.59	141.00 ± 1.82	0.252
Cl	106.35 ± 2.31	106.01 ± 2.09	0.286
Ca	2.44 ± 1.74	2.29 ± 0.11	0.351
TC	4.60 ± 0.96	5.13 ± 1.03	<0.001
TG	1.44 ± 0.67	1.63 ± 0.54	0.027
HDL	1.18 ± 0.20	1.30 ± 0.23	0.001
LDL	2.52 ± 0.66	2.69 ± 0.54	0.043
PT	12.98 ± 0.68	13.07 ± 0.56	0.328
APTT	36.50 ± 9.45	36.17 ± 3.08	0.714
FIB	2.81 ± 0.57	2.87 ± 0.63	0.470
TT	16.96 ± 0.79	16.83 ± 0.83	0.286
D-Dimer	0.30 ± 0.18	0.33 ± 0.27	0.330
FDP	1.71 ± 0.79	1.71 ± 0.92	0.943
CEA	5.86 ± 3.81	6.93 ± 3.04	0.031
SF	97.12 ± 47.94	114.98 ± 37.36	0.004
CA125	11.33 ± 5.07	13.09 ± 3.75	0.005
CA153	9.02 ± 4.37	10.51 ± 4.10	0.017
CA199	8.40 ± 3.91	10.49 ± 3.80	<0.001

WBC, White blood cell; NEUT, Neutrophil; LYM, Lymphocyte; MONO, Monocytes; AST, Aspartate aminotransferase; ALT, Alanine aminotransferase; ALB, Albumin; GLB, Globulin; ALP, Alkaline phosphatase; GGT, γ-glutamyl-transferase; CK, Creatine Kinase; LDH, Lactatedehydrogenase; CKMB, Creatine Kinase MB Form; α-HBDH, α-Hydroxybutyratedehydrogenase; CRE, Creatinine; K, Potassium; Na, Sodium; Cl, Chloride; Ca, Calcium; TC, Total Cholesterol; TG, Triglyceride; HDL, High density lipoprotein cholesterol; LDL, Low-Density Lipoprotein Cholesterol; PT, Prothrombin time; APTT, Activated partial thromboplastintime; FIB, Fibrinogen; TT, Thrombin time; FDP, Fibrin/Fibrinogen Degradation Products; CEA, Carcinoembryonic antigen; SF, Serum ferritin; CA125, Carbohydrate antigen 125; CA153, Carbohydrate antigen 153; CA199, Carbohydrate antigen 199.

### 3.2 Univariate and multivariate logistic regression analyses

Univariate logistic regression analysis demonstrated that white blood cell count (WBC), lymphocyte count (LYM), α-HBDH, total cholesterol (TC), triglycerides (TG), high-density lipoprotein (HDL), CEA, SF, CA125, CA153, and CA199 were significantly associated with lesion localization in early-stage NSCLC (**Table 4**).

**Table 4:** Analysis of univariate logistic regression.

Variables	OR (95%CI)	P-value
WBC	1.45 (1.17, 1.79)	0.001
LYM	2.08 (1.22, 3.54)	0.007
α-HBDH	1.02 (1.01, 1.03)	0.005
TC	1.71 (1.26, 2.32)	0.001
TG	1.61 (1.02, 2.54)	0.042
HDL	11.4 (2.89, 45.3)	0.001
LDL	1.58 (0.98, 2.52)	0.058
CEA	1.09 (1.00, 1.18)	0.045
SF	1.01 (1.00, 1.02)	0.008
CA125	1.08 (1.02, 1.15)	0.014
CA153	1.08 (1.01, 1.15)	0.021
CA199	1.14 (1.06, 1.23)	0.001

**Figure 1:**
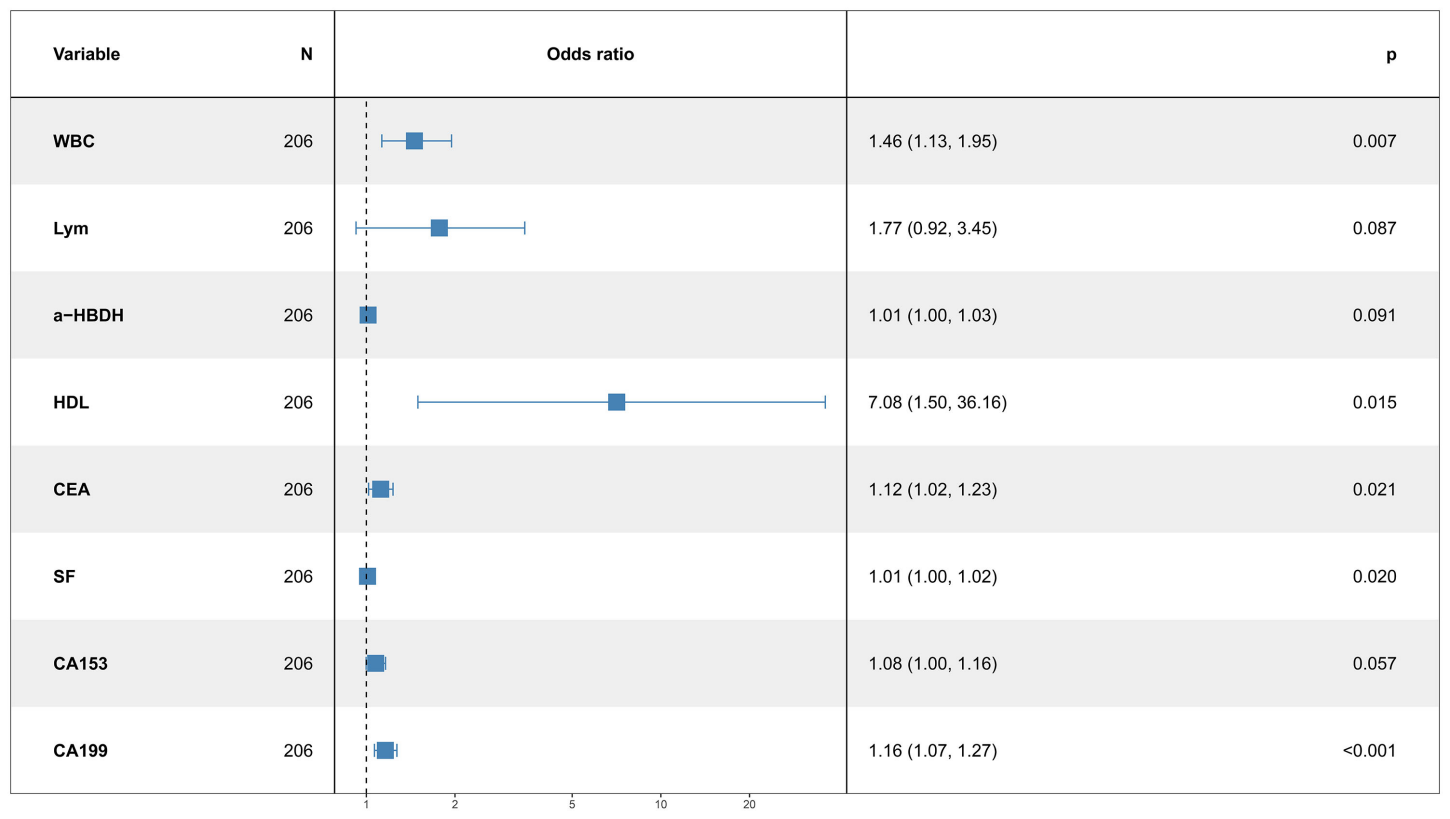
Multifactor logistic regression.

Multivariate logistic regression revealed that WBC (OR: 1.46, 95% CI: 1.13–1.95, *p* = 0.007), a-HBDH (OR: 1.01, 95% CI: 1.00–1.03, *p* = 0.041), HDL (OR: 7.08, 95% CI: 1.50–36.16, *p* = 0.015), CEA (OR: 1.12, 95% CI: 1.02–1.23, *p* = 0.021), SF (OR: 1.01, 95% CI: 1.00–1.02, *p* = 0.020), CA153 (OR: 1.08, 95% CI: 1.00–1.16, *p* = 0.037), and CA199 (OR: 1.16, 95% CI: 1.07–1.27, *p* < 0.001) were independent risk factors for tumor localization in the lower lobes (**Figure 1**).

### 3.3 Construction of a nomogram model for predicting lesion localization in early-stage NSCLC

**Figure 2:**
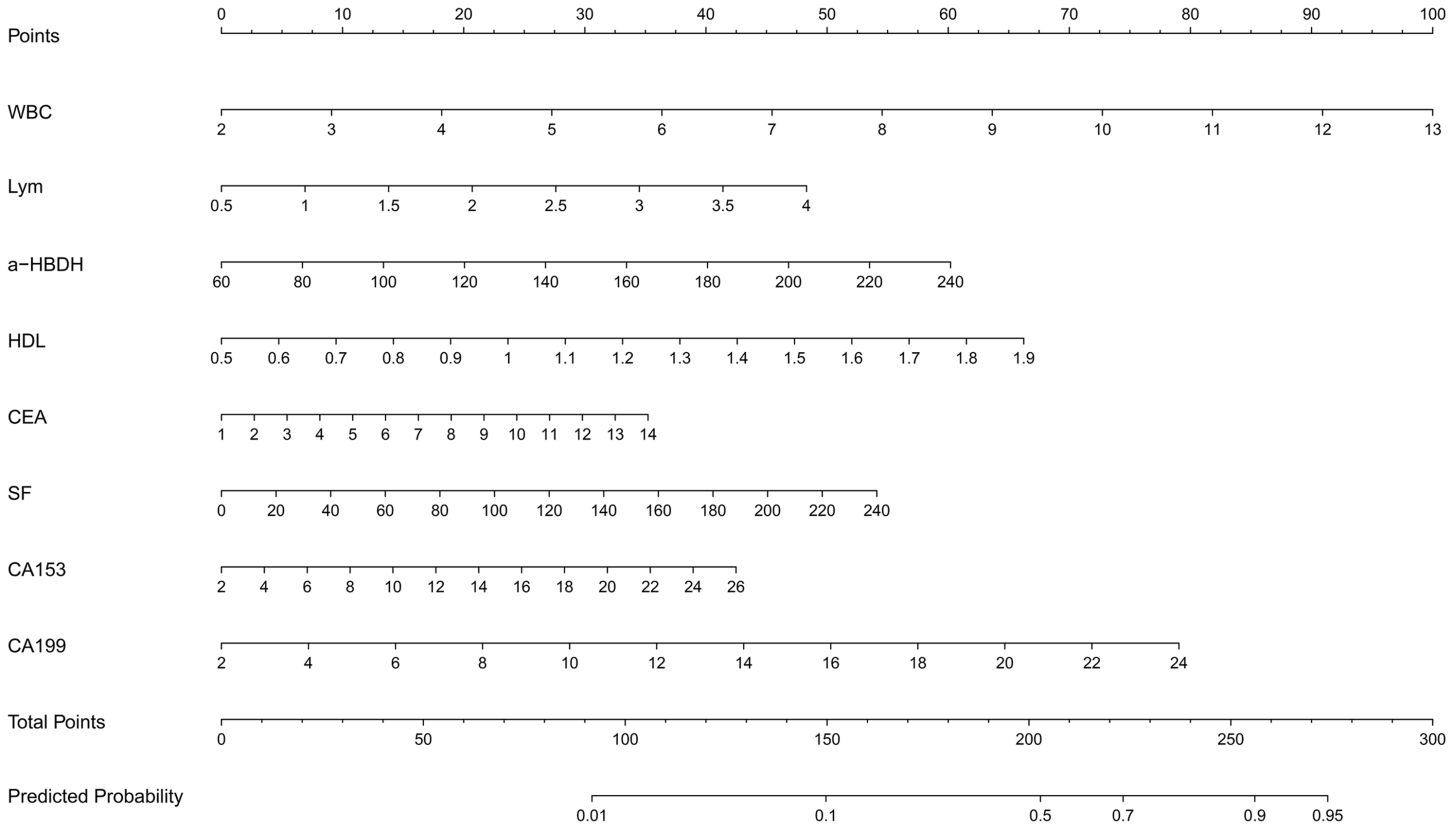
Nomogram.

A nomogram was developed to predict lesion localization in early-phase non-small cell lung cancer, using independent risk factors from multivariate logistic regression (**Figure 2**). WBC, LYM, α-HBDH, HDL, CEA, SF, CA153, and CA199 were included as predictors of increased risk for lower lobe lesion localization. A higher nomogram score indicated a greater probability of tumor localization in the lower lobes.

### 3.4 Evaluation of the nomogram model

**Figure 3:**
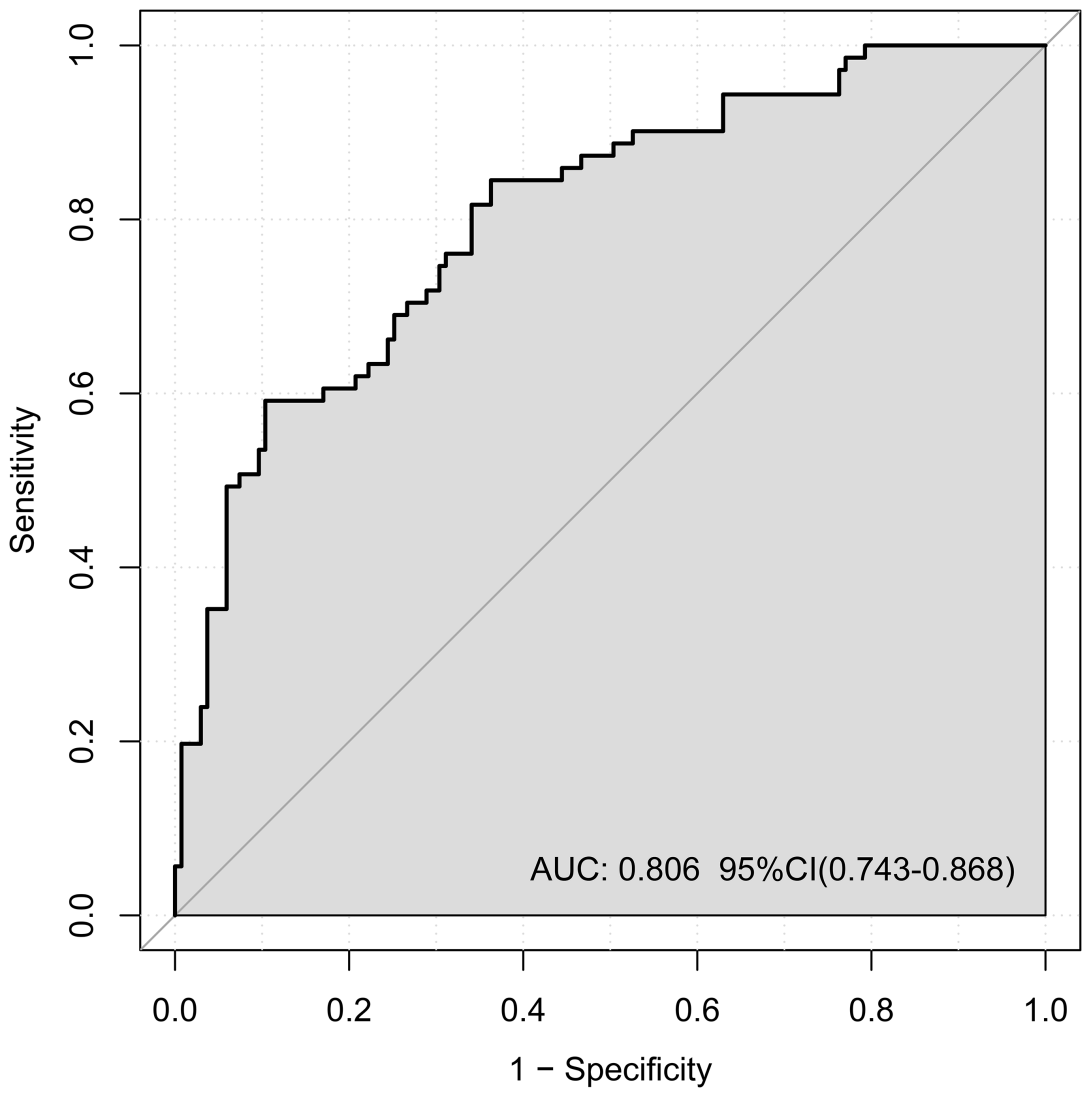
Receiver operating characteristic (ROC) curve of the nomogram.

ROC curve analysis was employed to assess the performance of the nomogram model (**Figure 3**). The AUC was 0.806 (95% CI: 0.743–0.868), and the model showed 89.2% sensitivity and 79.2% specificity, indicating good discriminative ability for predicting lesion localization in early-stage NSCLC.

**Figure 4:**
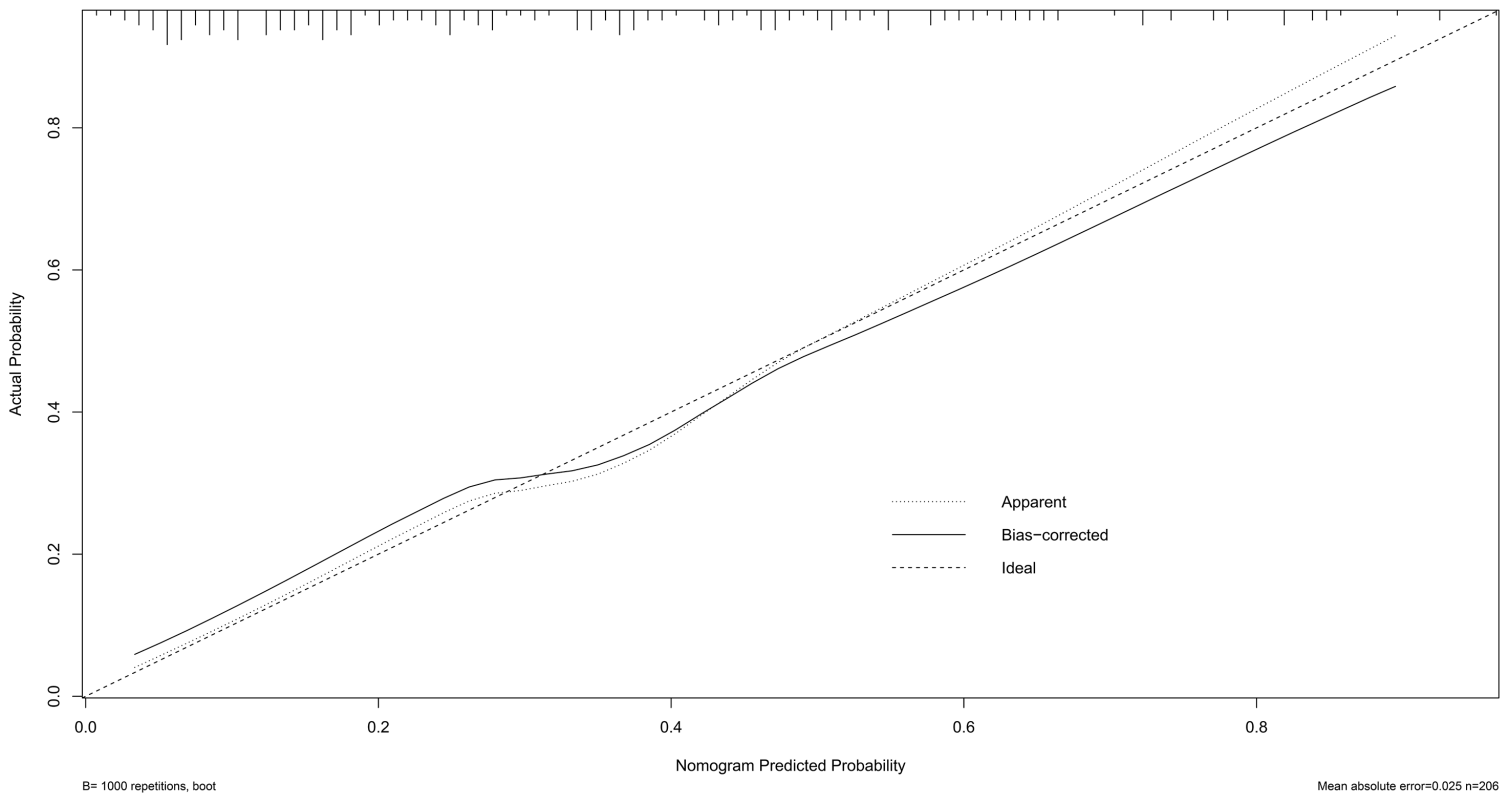
Calibration curve of the nomogram.

Calibration of the nomogram was performed using 1,000 bootstrap resamples (**Figure 4**). The dashed diagonal line represents the ideal reference for perfect prediction, while the solid line shows the model’s actual performance. The closer the two lines, the better the predictive accuracy. The absolute error between the predicted and actual curves was 0.025, indicating strong calibration and high agreement.

**Figure 5:**
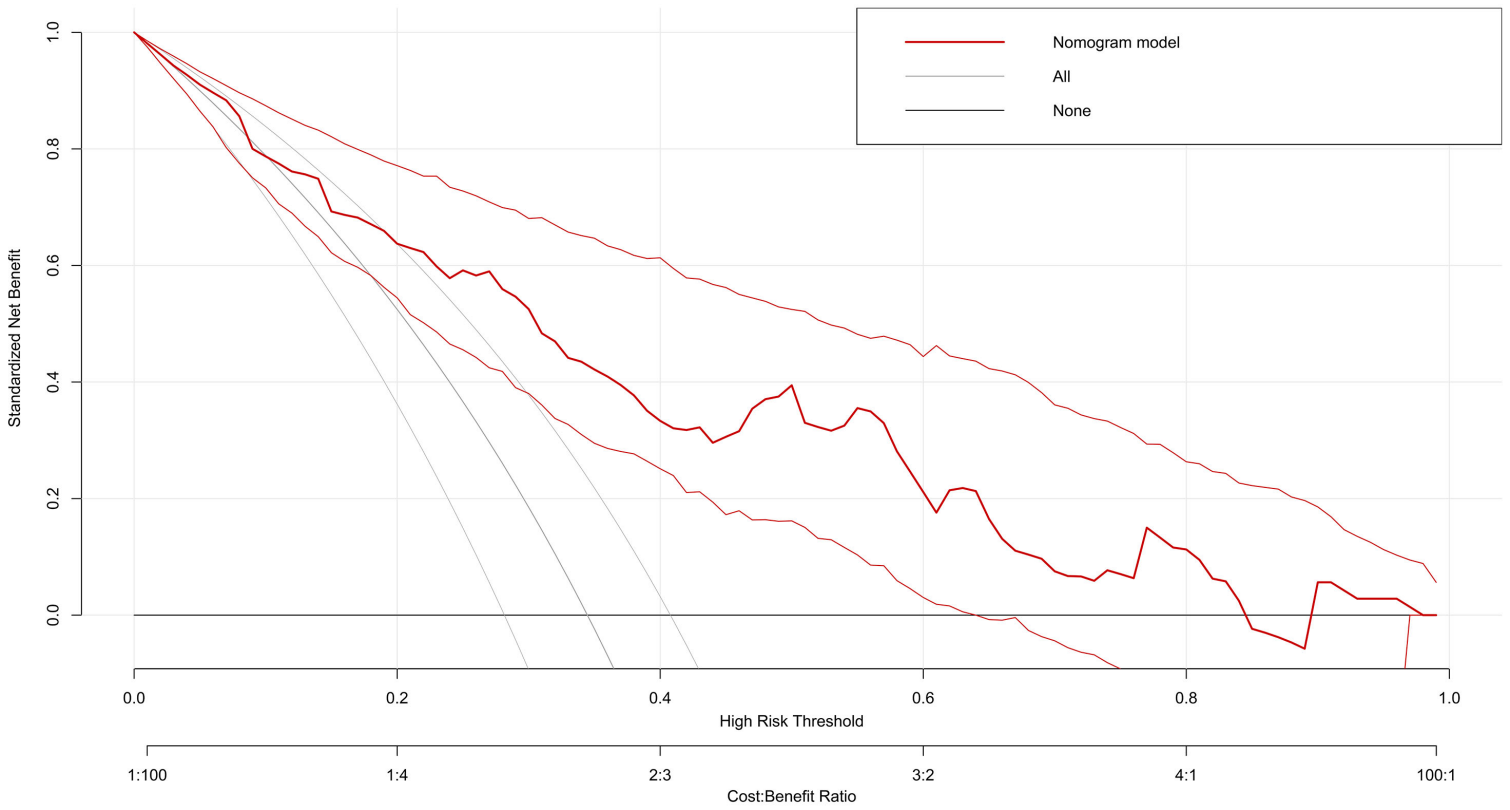
Decision curve analysis.

Decision Curve Analysis showed that the nomogram model provided favorable clinical benefit across a wide range of threshold probabilities (5 to 85%), supporting its importance in clinical decision-making (**Figure 5**).

## 4 Discussion

Tumor localization in NSCLC is closely linked to disease progression and prognosis. Primary tumor location is a prognostic factor in several malignancies, including esophageal, colorectal, and NSCLC (10, 11). A Japanese study found a significant association between NSCLC recurrence and lower lobe tumors (12). Lee et al. reported that NSCLC individuals with tumors outside the lower lobes had better 5-year survival rates after surgical resection than those with lower lobe tumors (13). Multiple studies show that NSCLC patients with lower lobe tumors have worse outcomes than those with upper lobe tumors (14–19). Thus, early identification of tumor localization in NSCLC is clinically valuable.

This study identified WBC, LYM, α-HBDH, HDL, CEA, SF, CA153, and CA199 as key factors associated with lower lobe lesion localization in early-stage NSCLC. Physiologically, the lower pulmonary regions exhibit a relatively greater degree of vascular perfusion compared to the upper zones. This relative hyperperfusion may predispose to more aggressive tumor behavior and contribute to the higher incidence of primary carcinomas originating in the lower lobe (20). Chronic inflammation is a well-established risk factor for cancer development (21). Furthermore, evidence suggests that elevated total white blood cell and neutrophil (NEU) counts are positively associated with an increased risk of both lung cancer and benign pulmonary diseases (22). Elevated WBC count reflects systemic inflammation, a major contributor to tumor progression, promoting tumor cell proliferation, invasion, and metastasis (23). Neutrophil accumulation enhances the inflammatory microenvironment, promoting tumor invasiveness (24). Lower lobe NSCLC tumors are more likely to complicate obstructive pneumonia, with neutrophilic infiltration further elevating WBC levels (25). Both lymphocyte percentage (LYM%) and red cell distribution width (RDW) have been demonstrated as independent predictors of lung cancer risk, with marked variations in LYM% observed across different histological subtypes of pulmonary carcinoma (26). α-HBDH, an isoenzyme of LDH, serves as a marker of glycolytic activity, reflecting the Warburg effect. Tumor cells upregulate LDH/a-HBDH to convert pyruvate to lactate, creating an acidic microenvironment that promotes invasion and metastasis (27). Previous studies by Chinese researchers have shown a strong link between α-HBDH levels and lung cancer development, progression, and prognosis. α-HBDH was identified as an independent prognostic factor for overall survival (OS) in patients with lung cancer. The high α-HBDH group exhibited a significantly reduced median OS of 6.4 months (95% CI 0.2–26.0) compared to the normal α-HBDH group (12.7 months, 95% CI 0.8–46.4), a difference that reached statistical significance (P = 0.023) (28). This may result from the hypoxic microenvironment in the lower lobes, which promotes glycolysis by upregulating LDH-A expression via hypoxia-inducible factor-1α (HIF-1α), accelerating tumor growth. Although no consensus exists on the link between tumor location (upper vs. lower lobe) and HDL-C levels in NSCLC patients, studies and mechanistic insights provide preliminary clues. Evidence from epidemiological studies indicates a J-shaped association between HDL levels and cancer mortality (p for nonlinearity < 0.01), with the lowest risk observed at HDL-C concentrations ranging from 64 to 68 mg/dL (**Figure 5**). For each 10 mg/dL increment in HDL-C beyond this range, the pooled risk ratio for cancer mortality was 1.02 (95% CI 0.98, 1.05) and 1.11 (95% CI 1.09, 1.14), respectively, compared to the reference interval (29). A case-control study conducted in China identified a strong link between low HDL-C levels and elevated NSCLC risk. The reduction in HDL-C may result from the increased cholesterol demand for tumor growth and membrane biosynthesis (30). HDL-C promotes cholesterol accumulation in tumor cell membranes, enhancing epidermal growth factor receptor (EGFR) signaling—an effect more commonly seen in adenocarcinomas in the lower lobes (31, 32).

Tumor markers are linked to tumor cell proliferation and differentiation. Dou et al. found that the median CEA level in lung adenocarcinoma patients was 6.6 ng/mL, confirming CEA as an independent predictor of lung adenocarcinoma (OR = 1.50, 95% CI: 1.04–2.16, p < 0.001) (33). A clinical investigation demonstrated that serum levels of CEA, SCC-AG, CYFRA 21-1, NSE, and CA199 exhibit a positive correlation with both tumor metastasis and disease staging (34). Lower lobe tumors are frequently associated with chronic inflammation, stimulating CEA secretion. Elevated SF levels release free iron (Fe^2+^), generating ROS through the Fenton reaction. These ROS cause DNA damage and activate matrix metalloproteinases (MMP-2/9), facilitating pleural invasion by enabling tumor cells to breach the pleural barrier—especially in lower lobe NSCLC tumors (35). This may be due to the anatomical proximity of the lower lobes to the diaphragm, where mechanical stress is more prominent, exacerbating pleural fissure formation through increased ROS generation. Previous studies identified SF as an independent factor associated with EGFR mutation status in NSCLC (36). In this study, CA153 and CA199 levels were significantly higher in patients with lower lobe NSCLC in relation to individuals with upper lobe tumors. Lower lobe tumors are more prone to pleural invasion, increasing the risk of poor prognosis. Gravitational effects cause secretions like mucus plugs to accumulate in the lower lobes, increasing susceptibility to chronic inflammation. This inflammation may further promote tumor aggressiveness and invasiveness. Previous studies have shown that both CA153 and CA199 are significantly associated with tumor development, progression, and poor prognosis (37, 38).Lower lobe tumors, particularly mucinous and adherent-type adenocarcinomas, often overexpress mucin1,the antigenic source of CA153, promoting its secretion (39). These tumors are more likely to invade the pleura, stimulating mesothelial cells to release CA153 (40). The hypoxic microenvironment may upregulate glycosyltransferases like fucosyltransferase 3 (FUT3) via HIF-1α, enhancing CA199 synthesis. A positive correlation between FUT3 mRNA expression and CA199 levels supports this mechanism (41).

This study is the first to combine inflammatory-lipid indices and tumor markers to predict the spatial localization of early-stage NSCLC lesions. It found significantly higher levels of WBC, LYM, α-HBDH, HDL, CEA, SF, CA153, and CA199 in lower lobe tumors compared to upper lobe tumors (all *p* < 0.05). These factors were independent predictors of lower lobe tumor localization. The research aims to provide a low-cost, dynamic diagnostic tool for clinical use. This predictive model could potentially be integrated with AI-driven systems in the future to achieve further refinement. AI-assisted interpretation may enhance its clinical applicability and strengthen its diagnostic or prognostic utility (42). However, this study is not without its limitations. First, the analysis focused on the upper and lower lobes, excluding the right middle lobe. Further investigations are needed to explore tumor localization in this region. Second, the study was conducted at the lobar level, thus necessitating further research involving more granular segmental-level analyses to refine spatial localization. Third, as a single-center retrospective study, the findings may be subject to selection bias and limited generalizability due to the absence of broader datasets. The study has strong clinical potential. Addressing the above revisions will enhance its robustness and generalizability. Further research should conduct large-scale, multi-center external validations to improve the generalizability of the model. Future studies should include larger, multicenter cohorts to refine the predictive model for more accurate spatial localization of early-stage NSCLC lesions.

## 5 Conclusion

This study confirms that white blood cell count, lymphocyte count, α-hydroxybutyrate dehydrogenase, high-density lipoprotein, carcinoembryonic antigen, serum ferritin, CA153, and CA199 are key predictors of tumor localization in non-small cell lung cancer. The nomogram model, built with these readily available clinical parameters, demonstrated strong predictive performance and offers a novel, low-cost tool for assessing early-stage NSCLC tumor distribution in clinical practice.

## Data Availability

The original contributions presented in the study are included in the article/supplementary material, further inquiries can be directed to the corresponding author.
